# The American Association of Obstetricians and Gynecologists

**Published:** 1888-09

**Authors:** 


### *THE AMERICAN ASSOCIATION OF OBSTETRICIANS AND GYNECOLOGISTS.*

Programme of the annual meeting to be held in the National Medical College building, H street, N. W., Washington, D. C., September 18, 19, and 20, 1888:

First Day—Tuesday, September 18th.

*Morning Session at 10 o'clock.*

Papers—"The Relations of the Abdominal Surgeon to the Obstetrician and Gynecologist," Dr. Albert Vander Veer, Albany. "Methods of Success in Abdominal Surgery," Mr. Lawson Tait, Birmingham, Eng. "Drainage in Abdominal and Pelvic Surgery," Dr. Joseph Price, Philadelphia. "Double Ovariotomy during Pregnancy;" a successful case going on to full term, Dr. William Warren Potter, Buffalo. "Vaginal Tamponnement in the Treatment of Prolapsed Ovaries," Dr. W. P. Manton, Detroit.

Adjournment at 1 P. M.

*Afternoon Session at 3 o'clock.*

"A Contribution to the Study of Pelvic Abscess," Dr. Clinton Cushing, San Francisco. "Treatment of Suppurative Peritonitis," Dr. Wm. H. Myers, Fort Wayne. "Laparotomy in Peritonitis," Dr. E. E. Montgomery, Philadelphia. "The Female Perineum: Its Anatomy, Physiological Function, and Methods of Restoration after Injury," illustrated with oxy-hydrogen light and screen, Dr. Henry O. Marcy, Boston. "The Surgical Treatment of the Perineum," Dr. Wm. H. Wathen, Louisville. "Ruptured Perineum," Dr. J. Henry Carstens, Detroit.

Adjournment at 5 P. M.

Second Day—Wednesday, September 19th.

*Business Session at 9 o'clock, for active Fellows only—Morning Session at 10 o'clock.*

Papers—"Some Points in Relation to the Diagnosis of Pregnancy in the Early Months," Dr. James P. Boyd, Albany. "A Contribution to the Study of the Neuroses of Pregnancy, with Report of a Case of Aphasia Graviditatis," Dr. G. A. Moses, St. Louis. "Treatment of Puerperal Convulsions," Dr. Melancthon Storrs, Hartford. "Heart Failure in the Puerperium," Dr. Thomas Lothrop, Buffalo. "The President's Address, "Dr. Wm. H. Taylor, Cincinnati.

Adjournment at 1 P. M.

*Afternoon Session at 3 o'clock.*

"Induced Labor," Dr. Byron Stanton, Cincinnati. "The Indications for Artificial Aid in Labor," Dr. Thomas Opie, Baltimore. "The Use and Abuse of Ergot in Obstetrical and Gynecological Practice," Dr. J. M. Dunham, Columbus. "Operative Treatment in Uterine Carcinoma," Dr. Geo. R. Shepherd, Hartford. "The Technique of Vaginal Hysterectomy," Dr. James H. Etheridge, Chicago.

Adjournment at 5 P. M.

THIRD DAY—THURSDAY, SEPTEMBER 20TH.

*Morning Session at 10 o'clock.*

"Uterine Fibroids: Their Diagnosis and Treatment," Dr. Thomas J. Maxwell, Keokuk. "Operation for an unusual Case of Subserous Uterine Fibroid, Dr. Hampton Eugene Hill, Saco. "Apostoli's Method in the Treatment of Fibroid Tumors," Dr. Franklin H. Martin, Chicago. "Tumors of the Abdominal Wall," Dr. Charles A. L. Reed, Cincinnati. "Desmoid (Fibroid) Tumors of the Abdominal Wall," Dr. Edward J. Ill, Newark. "The Reflexes Reflected; or, Some Things that Retard Progress in Gynecic Surgery," Dr. Joseph Eastman, Indianapolis.

Adjournment at 1 P. M.

*Afternoon Session at 3 o'clock.*

Discussion—"Extra-Uterine Pregnancy," Pathology, Dr. Franklin Townsend, Albany; Diagnosis, Dr. Joseph Price, Philadelphia; Treatment (*a*) Medical and Electrical, (*b*) Surgical, Mr. Lawson Tait, Dr. E. E. Montgomery, Dr. Charles A. L. Reed, Dr. Jos. Eastman, Dr. A. Vander Veer.

By Invitation—"A New Operation for the Repair of Lacerated Perineum," Dr. Bernard Burns, Allegheny City. I. "Some Cases of Puerperal Eclampsia;" II. "Endometritis Treated by Injection of Pure Nitric Acid," by Dr. A. Cordes, Geneva, Switzerland. "Some Minute but Important Details in the Use of the Continuous Current in Gynecology," Dr. A. Lapthorn Smith, Montreal. "Some Common Diseases of the Skin, associated with Sexual Disorders in the Female," Dr. George H. Rohé, Baltimore.

Final adjournment at 5 P. M.

OFFICERS FOR 1888.

President, William H. Taylor, Cincinnati. Vice-Presidents: E. E. Montgomery, Philadelphia; J. Henry Carstens, Detroit. Secretary, William Warren Potter, Buffalo. Treasurer, X. O. Werder, Pittsburgh. Executive Council: Thomas Opie, Baltimore; James H. Etheridge, Chicago; Clinton Cushing, San Francisco; Melancthon Storrs, Hartford; Byron Stanton, Cincinnati. Delegate-elect to the Executive Committee of the Congress of American Physicians and Surgeons, James P. Boyd, Albany; alternate, Hampton E. Hill, Saco, Me.

